# Nationwide External Quality Assessment of SARS-CoV-2 Molecular Testing, South Korea

**DOI:** 10.3201/eid2610.202551

**Published:** 2020-10

**Authors:** Heungsup Sung, Myung-Guk Han, Cheon-Kwon Yoo, Sang-Won Lee, Yoon-Seok Chung, Jae-Sun Park, Mi-Na Kim, Hyukmin Lee, Ki Ho Hong, Moon-Woo Seong, Kyunghoon Lee, Sail Chun, Wee Gyo Lee, Gye-Cheol Kwon, Won-Ki Min

**Affiliations:** University of Ulsan College of Medicine and Asan Medical Center, Seoul, South Korea (H. Sung, M.-N. Kim, S. Chun, W.K. Min);; Korea Centers for Disease Control and Prevention, Chungcheongbuk-do, South Korea (M.-G. Han, C.-K. Yoo, S.-W. Lee, Y.-S. Chung, J.-S. Park);; Yonsei University College of Medicine, Seoul (H. Lee);; Seoul Medical Center, Seoul (K.-H. Hong);; Seoul National University College of Medicine, Seoul (M.-W. Seong, K. Lee);; Ajou University School of Medicine, Suwon, South Korea (W.G. Lee);; Chungnam National University School of Medicine, Daejeon, South Korea (G.-C. Kwon)

**Keywords:** COVID-19, coronavirus disease, SARS-CoV-2, severe acute respiratory syndrome coronavirus 2, viruses, respiratory infections, zoonoses, external quality assessment, South Korea

## Abstract

External quality assessment (EQA) is essential for ensuring reliable test results, especially when laboratories are using assays authorized for emergency use for newly emerging pathogens. We developed an EQA panel to assess the quality of real-time reverse transcription PCR assays being used in South Korea to detect severe acute respiratory syndrome coronavirus 2 (SARS-CoV-2). With the participation of 23 public health organization laboratories and 95 nongovernmental laboratories involved in SARS-CoV-2 testing, we conducted qualitative and semiquantitative performance assessments by using pooled respiratory samples containing different viral loads of SARS-CoV-2 or human coronavirus OC43. A total of 110 (93.2%) laboratories reported correct results for all qualitative tests; 29 (24.6%) laboratories had >1 outliers according to cycle threshold values. Our EQA panel identified the potential weaknesses of currently available commercial reagent kits. The methodology we used can provide practical experience for those planning to conduct evaluations for testing of SARS-CoV-2 and other emerging pathogens in the future.

The current outbreak of coronavirus disease (COVID-19), caused by severe acute respiratory syndrome coronavirus 2 (SARS-CoV-2), continues to spread. As of June 27, 2020, the pandemic had affected 214 countries, resulting in 9,653,048 recorded cases and 491,128 deaths (*1*,*2*). Early detection of SARS-CoV-2 and immediate isolation of infected patients from the susceptible population is important for preventing the spread of infection (*3*). Real-time reverse transcription PCR (rRT-PCR) is currently the most reliable method for diagnosing COVID-19 (*4*).

Since March 2, 2020, the number of newly reported cases in South Korea appears to be declining; the mean number of daily new confirmed cases decreased to 40 by the end of June (*5*). Intensive SARS-CoV-2 testing helped to contain the spread of the disease in South Korea. Since March 1, South Korea (population »51 million) has performed »20,000 tests per day by using rRT-PCR. The outstanding achievements of the public health response were attributable to the rapid expansion of diagnostic testing capabilities resulting from the collaboration between the public and private sectors. In June 2016, South Korea enacted emergency use authorization (EUA) legislation with the aim of supplying commercial kits to meet the demands of nongovernmental clinical laboratories and to guarantee quality assurance through mandatory technical training regarding standardized laboratory guidelines and external quality assessment (EQA) (*6*).

As of March 31, after successfully completing a proficiency testing panel consisting of 7-plasmid DNA specimens, a total of 95 nongovernmental clinical laboratories were conducting SARS-CoV-2 tests by using 5 different EUA kits (*6*). However, the nucleic acid extraction methods, rRT-PCR reagents, and thermocyclers used differed among laboratories. EQAs using pooled respiratory samples spiked with inactivated cultured SARS-CoV-2 had indicated the possible effects of these variations on assay performance, thereby allowing the participating laboratories to assess the quality and identify the possible weaknesses and strengths of the currently used diagnostic methods (*7*,*8*). Therefore, we developed an EQA panel to assess the quality of SARS-CoV-2 rRT-PCR assays in South Korea.

## Methods

### Participants

Because participation in the nationwide EQA requested by the Korea Centers for Disease Control (KCDC) was mandatory for the 118 laboratories conducting SARS-CoV-2 tests, these laboratories were included in the our study by default. Because the study was a survey and an EQA that did not include personal identifiers or patient data, the requirement for institutional review board approval was waived (waiver no. AMC IRB 2020-0547). The upper and lower respiratory tract samples from SARS-CoV-2–negative patients were also included in the study.

### Specimen Preparation

To construct the SARS-CoV-2 proficiency test panel, Vero cells (ATCC CCL-81, American Type Culture Collection, https://www.atcc.org) were inoculated with the SARS-CoV-2 KNIH001 strain (from KCDC) and cultured at 37°C at 5% CO_2_ for 5 days (*9*). Infection was confirmed by assessing the cytopathic effects and by rRT-PCR assays using primers and probe sets described previously by Corman et al. (*10*). The titer was 1.0 × 10^6^ PFU/mL, and the cycle threshold (C_t_) value of the SARS-CoV-2 *E* gene was »16. The virus was inactivated at 70°C for 1 hour. No infectious virus was detected on testing for residual infectivity after heat treatment by inoculation in cell culture. Dilution matrices were established by using pooled nasopharyngeal aspirates, to represent upper respiratory tract samples, and pooled sputum or bronchoalveolar lavage fluids, to represent lower respiratory tract samples. All pooled samples were negative for common respiratory viruses and SARS-CoV-2. Liquillizer (MetaSystems, https://metasystems-international.com) was added to the pooled lower respiratory tract samples for dilution to achieve a final concentration of 10%.

The proficiency test panel included 4 each of upper and lower respiratory tract samples containing serial 10-fold dilutions of SARS-CoV-2–positive cell culture supernatant (1:2 × 10^2^−1:2 × 10^5^), 1 human coronavirus OC43 (HCoV-OC43)–positive/SARS-CoV-2–negative upper respiratory tract sample, and 1 negative lower respiratory tract sample. The samples were immediately frozen at −70°C after aliquoting. Although participants were informed that the materials were nonbiohazardous, we recommended that they be handled according to general basic requirements regarding human specimens.

### Validation and Dispatch of Panel Tests and Collection of EQA Results

To validate stability and homogeneity, EQA panel samples were assayed by 3 extraction systems, 4 EUA reagents, and 2 thermocyclers. For assessing homogeneity, 3 panels were selected at random and assayed in triplicates, generating 9 test results; for stability, samples were maintained at −70°C for 1 day, 3 days, and 7 days. Three panels from each storage condition were then selected at random and assayed 3 times. EQA samples were shipped on dry ice with temperature monitoring by Green Cross Labcell (http://www.gclabcell.co.kr). Delivery to all laboratories, including those on Jeju Island, was completed within 10 hours. The Korean Association of External Quality Assessment Service (KEQAS) was responsible for the transport of the EQA material, collection of the EQA results, and evaluation of results. All EQA data, including those of test sample volumes, extraction reagents and instruments, and elution volumes, were submitted through the KEQAS program web site (http://eqas.keqas.org).

### Evaluating the EQA Results and Statistical Analysis

For qualitative evaluations, only samples with an >80% agreement rate compared with the expected results were evaluated (*11*). All EUA kits included the manufacturer’s instructions for threshold settings or use of an exclusive interpretation–viewer program from the manufacturer. For evaluations of semiquantitative data, the mean, median, and interquartile ranges of the C_t_ values were converted to box-and-whisker plots. Outliers were defined when determined by a double-sided Grubbs test, when a negative result for positive sample was given, or when any C_t_ value in negative samples was reported. The outlier frequencies were compared by using the χ^2^ test. The likelihood ratios for different tests were calculated from a 2 × k table. For homogeneity and stability tests, the mean value and percentage coefficient of variation (%CV) of the 9 results from triplicated assays of 3 samples were analyzed, and assumption of homogeneity of variance was tested by using Levine’s test of equality of variances. Homogeneity and stability were satisfactory when the %CV was <5 and p value was >0.05. MedCalc Statistical Software version 19.2.1 (MedCalc Software Ltd, https://www.medcalc.org) was used for all statistical analyses.

## Results

### Participating Laboratories

Twenty-three public laboratories conducting COVID-19 tests, including 18 regional Institutes of Health and Environment, 4 National Quarantine Stations, and 1 Armed Force Medical Science Research Institute, participated, along with 95 nongovernmental clinical laboratories. Sixty-six (55.9%) laboratories were located in the Seoul, Incheon, and Gyeonggi metropolitan areas, in which 49.6% of the total population of South Korea resides.

### SARS-CoV-2 Testing Protocols

**Figure 1:**
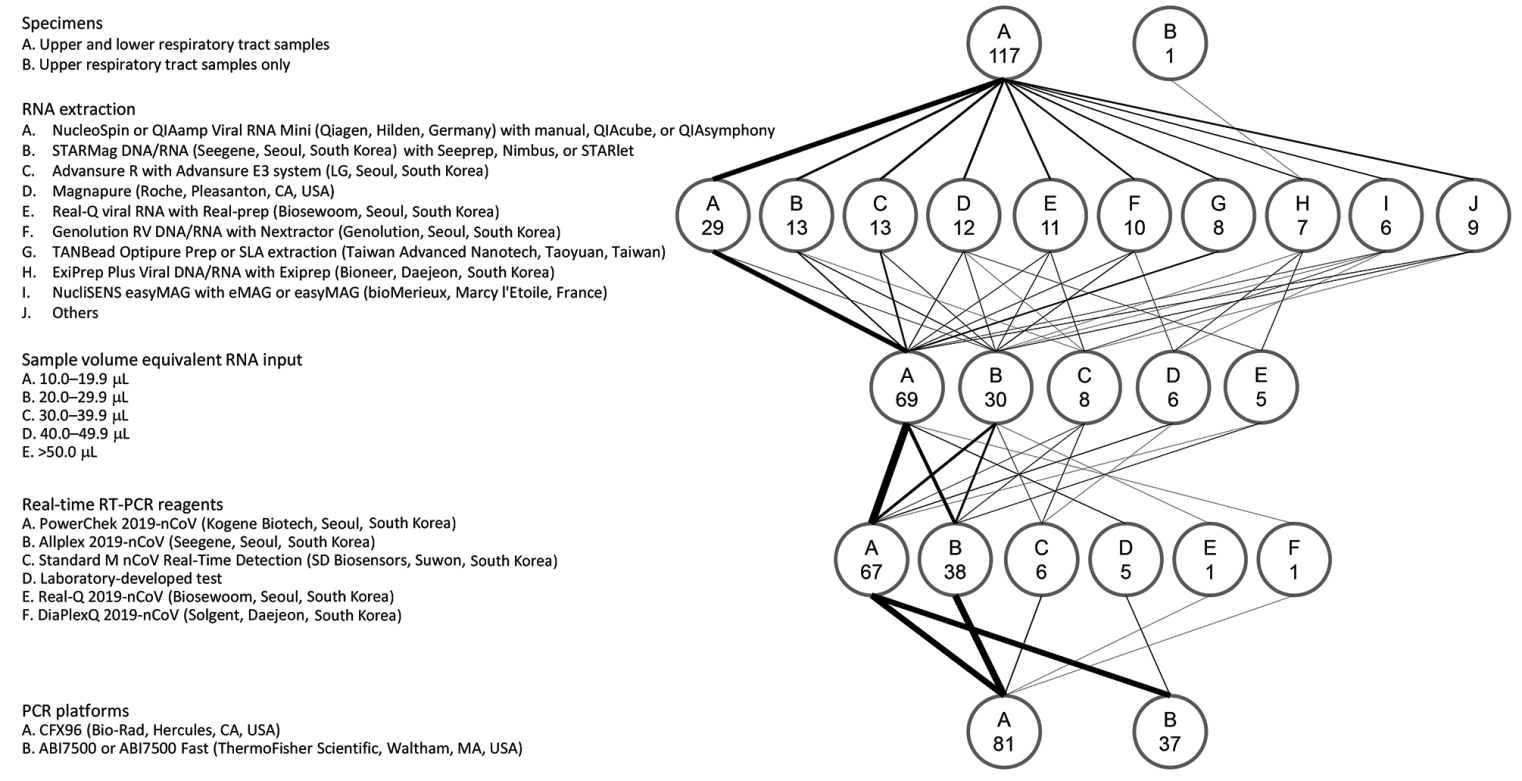
Protocols used for real-time RT-PCR in 118 laboratories participating in an external quality assessment of severe acute respiratory syndrome coronavirus 2 testing, South Korea, March 23–27, 2020. The flow diagram shows the variations in specimens tested, RNA extraction platforms, PCR reagents and amplification platforms, and sample volume equivalent RNA input used in the PCR reaction. The weight of the lines reflects the number of laboratories using a particular step. Numbers in the circles indicate number of laboratories. RT-PCR, reverse transcription PCR.

**Figure 2:**
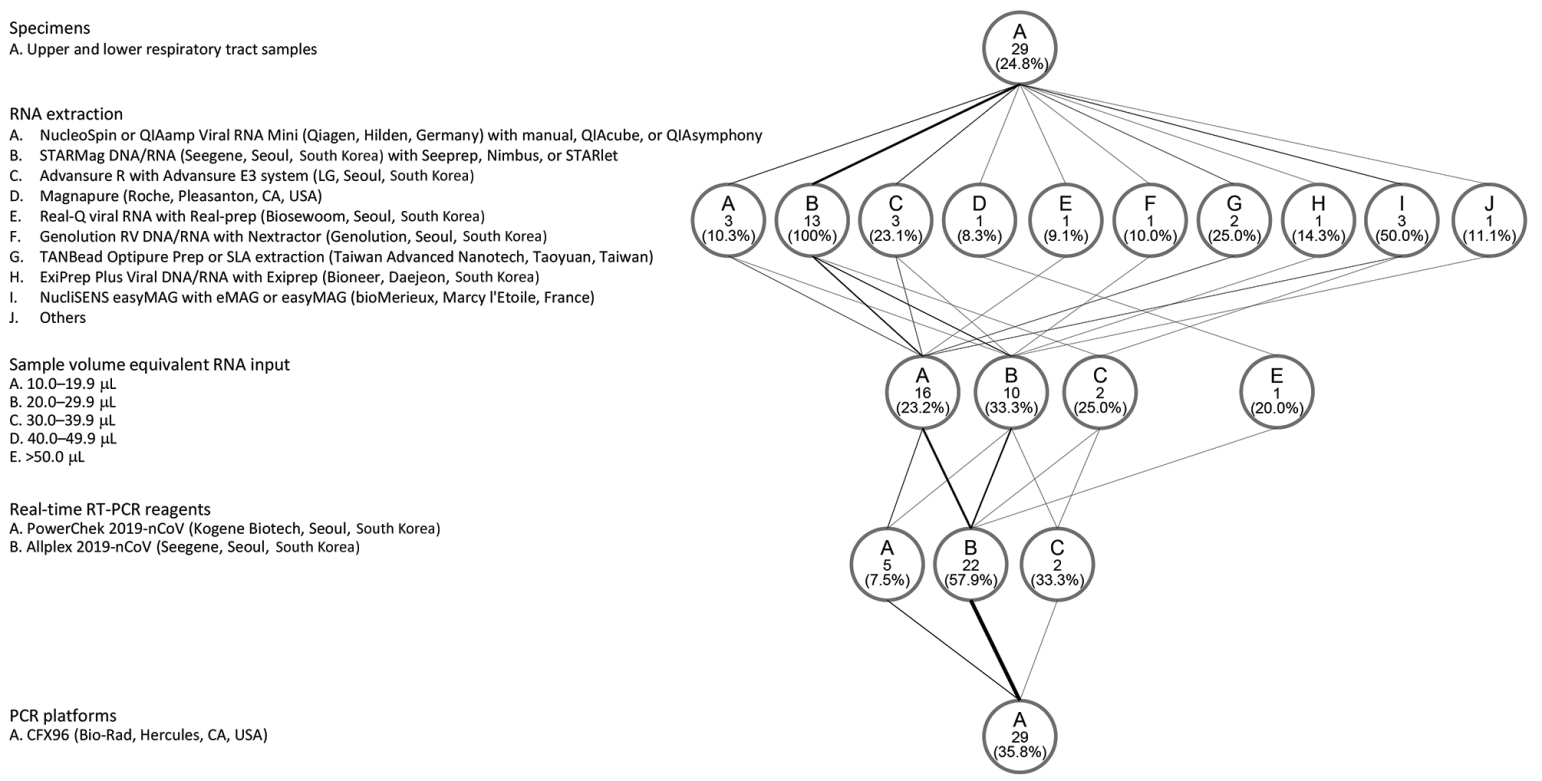
Protocols used for laboratories that reported >1 outliers in results of real-time RT-PCR tests for severe acute respiratory syndrome coronavirus 2, South Korea, March 23–27, 2020. The flow diagram shows the variations in specimens tested, RNA extraction platforms, PCR reagents and amplification platforms, and sample volume equivalent RNA input used in the PCR reaction. The weight of the lines reflects the number of laboratories using a particular step. Numbers in the circles indicate number of laboratories. RT-PCR, reverse transcription PCR.

Protocols for SARS-CoV-2 rRT-PCR varied among the 118 participating laboratories (Figure 1). Ninety-five nongovernmental clinical laboratories were allowed to use only 1 of the 5 EUA rRT-PCR reagents. Five regional Institutes of Health and Environment used laboratory-developed tests using the primers and probes described by Corman et al. (*10*) and AgPath-ID 1-step RT-PCR reagents (ThermoFisher Scientific, https://www.thermofisher.com). A large variance in extraction steps, reagents, instruments, sample volumes, and elution volumes (i.e., sample volume equivalent RNA input) was observed (Figures 1, 2). For SARS-CoV-2 detection, 67 laboratories (56.8%) used the PowerChek 2019-nCoV Kit (Kogene Biotech, http://www.kogene.co.kr), which was the first EUA SARS-CoV-2 assay cleared by KCDC and the Korea Food and Drug Administration, and 38 laboratories (32.2%) used the Allplex 2019-nCoV Kit (Seegene, http://www.seegene.com).

### Qualitative rRT-PCR Results

Because the lower respiratory tract sample with the highest dilution (nCoV-2041) showed 78.8% (93/118) agreement compared with the expected results, lower respiratory tract sample results were excluded from the final analysis. A total of 110 laboratories (93.2%) reported correct results for all qualitative tests.

Among the 38 laboratories using the Allplex 2019-nCoV Kit (Seegene), 8 (21.1%) laboratories had >1 incorrect results, in which all incorrect results were reported from lower respiratory tract samples (nCoV-20–41, −42, −43, or −45) (Appendix). Eighty laboratories using the other EUA kits or WHO primers and probes provided correct results for all evaluable results. The likelihood ratio of unacceptable results from the Allplex 2019-nCoV Kit compared with the other kits was 0.273 (95% CI 0.201−0.370). Other kits’ 95% CIs of likelihood ratios included 1.000. Extraction reagent kits used by laboratories that reported incorrect results were STARMag DNA/RNA Extraction Kit (Seegene) and NucliSENS easyMAG Extraction Kit (bioMérieux, https://www.biomerieux.com) used by 3 laboratories. The other 2 laboratories used Advansure R Extraction Kit (LG Chem, https://www.lgchem.com) or Exiprep Plus Viral DNA/RNA Extraction Kit (Bioneer, https://eng.bioneer.com).

### Semiquantitative Results by C_t_ value

**Figure 3:**
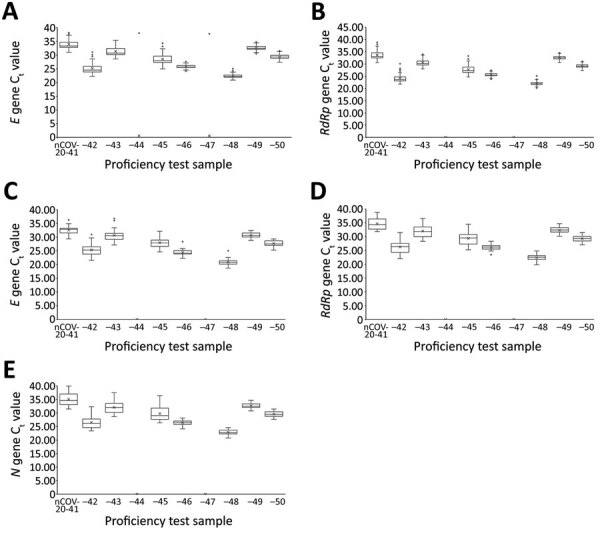
Semiquantitative real-time reverse transcription PCR C_t_ values for severe acute respiratory syndrome coronavirus 2 proficiency panel samples tested by PowerChek and Allplex 2019-nCoV kits, South Korea, March 23–27, 2020. Horizontal line within each box denotes the median value; x indicates the mean; top and bottom of box indicate third and first quartiles, respectively; error bars indicate minimum and maximum values; dots indicate outlier results. *E* gene (A) and *RdRp* gene (B) C_t_ values were from 67 laboratories using the PowerChek 2019-nCoV reagents; *E* gene (C), *RdRp* gene (D), and *N* gene (E) C_t_ values were from 38 laboratories using the Allplex 2019-nCoV reagents. C_t_, cycle threshold.

The C_t_ values of the SARS-CoV-2 *RdRp* gene for each EUA assay, except for the DiaPlexQ 2019-nCoV kit (Solgent, http://www.solgent.com), which detects the *ORF1a* and *N* genes, and expected C_t_ value for each sample for PowerChek 2019-nCoV and Allplex 2019-nCoV are shown in Table 1. *E* gene and *RdRp* gene C_t_ values from 67 laboratories using the PowerChek 2019-nCoV reagents and *E* gene, *RdRp* gene, and *N* gene C_t_ values from 38 laboratories using the Allplex 2019-nCoV reagents are shown in Figure 3.

**Table 1:** Test results for severe acute respiratory syndrome coronavirus 2 *RdRp* gene obtained from the proficiency test provider (expected value), Asan Medical Center, Seoul, and from participating laboratories according to the reagent used, South Korea, March 23–27, 2020*

Sample no.	Dilution	PowerChek			Allplex		Standard M, N = 6	Laboratory-developed test, N = 5	Real-Q, N = 1
		Expected value	Participating laboratories, N = 67		Expected value	Participating laboratories, N = 38†			
41	1:2 × 10^5^	33.60	33.64 + 1.73		34.37	34.64 + 2.23‡	30.38 (29.44–35.46)§	34.32 (32.57–34.62)	38.27
42	1:2 × 10^2^	24.62	24.16 + 1.68		25.67	26.29 + 2.34	21.28 (19.98–27.54)	25.94 (23.28–28.07)	24.06
43	1:2 × 10^4^	30.73	30.60 + 1.45		31.34	32.05 + 2.43¶	27.49 (26.79–33.39)	31.66 (30.01–32.83)	30.54
45	1:2 × 10^3^	27.69	27.71 + 1.66		28.73	29.40 + 2.51#	24.67 (23.51–31·21)	28.12 (26.77–29.05)	27.31
46	1:2 × 10^3^	24.82	25.61 + 0.76		26.12	26.07 + 1.11	22.18 (21.50–23.46)	27.78 (25.74–28.49)	26.61
48	1:2 × 10^2^	21.27	22.06 + 0.85		22.49	22.41 + 1.06	19.06 (17.20–19.86)	24.07 (22.04–25.02)	22.96
49	1:2 × 10^5^	32.14	32.57 + 0.96		32.55	32.35 + 1.13	29.36 (27.49–30.02)	34.39 (32.18–35.42)	33.36
50	1:2 × 10^4^	28.58	29.19 + 0.83		29.45	29.32 + 1.10	25.82 (24.20–27.01)	31.29 (29.38–32.40)	29.21

*Cycle threshold values are shown. Mean + SD is shown for the results obtained by using the PowerChek 2019-nCoV and Allplex 2019-nCoV kits. Median and range are shown for the results obtained by using the Standard M nCoV-Detection and laboratory-developed tests. †For nCoV-20–41−45, the number of laboratories was 37. ‡Calculated after exclusion of negative results from 9 laboratories. §Calculated after exclusion of negative results from 1 laboratory. ¶Calculated after exclusion of negative results from 3 laboratories. #Calculated after exclusion of negative results from 1 laboratory.

For extraction kits, all 13 laboratories using the STARMag DNA/RNA extraction kit had >1 outlier results (Figure 2). The likelihood ratio of outliers from the STARMag DNA/RNA kit compared with the other kits was 0.000 (95% CI 0.000−0.205). Other extraction kits’ 95% CIs of likelihood ratios included 1.000.

Among the laboratories using the PowerCheck 2019-nCoV kit, five (0.7%, 5/670) outliers occurred in the *E* gene C_t_ values at 5 (7.5%) laboratories. Three (0.4%, 3/670) outliers occurred in the *RdRp* gene C_t_ values from 2 (3.0%) laboratories (Appendix).

Among the laboratories using the Allplex 2019-nCoV kit, 37 (9.9%) outliers occurred in *E* gene results, 13 (3.5%) in *RdRp* gene results, and 5 (1.3%) in *N* gene results (Appendix). The frequency of total outliers for the Allplex 2019-nCoV kit was significantly higher than those for the PowerChek 2019-nCoV kit (4.9% vs. 0.6%; p<0.0001). Except for 2 outliers for the *E* gene, all other outliers were negative results for lower respiratory samples that should have been positive. Among the laboratories using the Standard M nCoV Real-Time Detection kit (SD Biosensor, http://www.sdbiosensor.com), 2 laboratories (33.3%) that used the STARMag extraction kit reported negative results for the nCoV-20–41 sample.

### Variation by Extraction Method and rRT-PCR Reagent of EQA samples

For the Standard M nCoV Real-Time Detection kit, the *E* gene C_t_ value was lower, by »2.8, than that of the Allplex nCoV-2019 kit and lower, by »3.9, than that of the PowerChek nCoV-2019 kit. For the Real-Q viral RNA extraction kit, the *E* gene C_t_ value was lower, by »1.4, than that of the NucliSENS easyMAG extraction kit. For the STARMag DNA/RNA extraction kit, 1 negative result occurred for the nCoV-20–41 *E* gene. However, the results were positive for the *RdRp* and *N* genes using the Allplex nCoV-2019 kit.

### Homogeneity and Stability of EQA Samples

For homogeneity tests, the mean C_t_ value and %CV of each target gene using the NucliSENS easyMAG kit for extraction and the Allplex nCoV-2019 kit for amplification are shown in Table 2. All the C_t_ values were within acceptable ranges, with %CVs <5. For stability tests, C_t_ values obtained for the SARS-CoV-2 EQA panel were also within acceptable ranges, with %CVs <5 (data not shown).

**Table 2:** Severe acute respiratory syndrome coronavirus 2 external quality assessment panel homogeneity tests of triplicate test results of 3 samples using the NucliSENS easyMAG extraction and Allplex nCoV-2019 kits performed at the Asan Medical Center, Seoul, South Korea, as a proficiency test provider, March 23, 2020*

Sample no.	*E* gene			*RdRp* gene			*N* gene	
	Mean C_t_	%CV		Mean C_t_	%CV		Mean C_t_	%CV
41	33.82	4.5		34.37	1.6		34.38	0.5
42	25.15	1.4		25.67	1.8		26.67	0.5
43	30.33	1.5		31.34	1.5		31.55	0.7
44	ND	ND		ND	ND		ND	ND
45	27.52	2.2		28.73	0.3		29.05	0.7
46	24.47	0.7		26.12	0.3		27.05	0.2
47	ND	ND		ND	ND		ND	ND
48	20.68	2.5		22.49	0.6		23.49	0.3
49	31.07	0.8		32.55	0.6		33.32	0.9
50	27.89	0.9		29.45	0.2		30.35	0.6

*C_t_, cycle threshold; EQA, external quality assessment; %CV, percentage coefficient of variation; ND, not detected.

## Discussion

The most important element for reducing the transmission of SARS-CoV-2 is the early detection of SARS-CoV-2 in patients. Shortening the time to diagnosis could substantially reduce the risk for transmission of SARS-CoV-2 (*12*). Performing diagnostic tests for newly emerging pathogens, such as SARS-CoV-2, exclusively in governmental and public laboratories substantially increases the delay resulting from logistics and analytical processes. As a result, diagnosis and isolation of patients, contact tracing, and even treatment of symptomatic patients can be delayed. Thus, rapid extension of diagnostic testing for emerging pathogens to nongovernmental clinical laboratories has many advantages, such as shorter turnaround times (*13*). To ensure reliable test results, EQA is a fundamental element, especially when using EUA diagnostic kits for newly emerging pathogens (*8*,*14*). The EQA we describe is unique because of its nationwide scale and because it included public and nongovernmental clinical laboratories conducting SARS-CoV-2 testing. This EQA was well-timed to support the laboratory responses to minimize the ongoing outbreak. All public and nongovernmental laboratories conducting SARS-CoV-2 molecular diagnostics reported their results in this nationwide EQA assessment in South Korea. The performance of the participants was good; overall accuracy was 100% for upper respiratory tract samples and 93.2% for lower respiratory tract samples.

On the basis of the laboratory results, the nCoV-20–41 sample was omitted from qualitative analysis. Initially, cultured SARS-CoV-2 were heat-inactivated at 65°C for 30 minutes (*15*). However, the second passage of cell cultures with inactivated viruses showed equivocal cytopathic effects, and therefore, the cultured viruses were further heat-inactivated at 70°C for 1 hour. The relatively high temperature and prolonged heat inactivation might have caused viral capsid denaturation and release of RNA. Viral RNA in the lower respiratory samples are vulnerable to degradation by RNase because of the lack of preservatives, in contrast to that observed in the universal transport medium used for upper respiratory tract samples. C_t_ values of the lower respiratory tract samples might have been higher than those of the upper respiratory tract samples, despite aliquoting the same amount of virus. Including replicate samples using the same matrix for assessing test consistency is preferred.

Among 118 public and nongovernment clinical laboratories, 8 (6.8%) laboratories using the Allplex 2019-nCoV kit reported >1 incorrect results for lower respiratory tract samples in the qualitative assessment. Whether this finding was attributable to the matrix effects of the proficiency test samples or the decreased sensitivity of the Allplex 2019-nCoV kit is unclear. Because the Liquillizer mucolytic agent was added to the inactivated virus-spiked lower respiratory tract samples, it could have adversely affected the extraction or the amplification procedures, although no interference with molecular methods has been observed by the manufacturer (*16*). Laboratories that had incorrect results on their qualitative tests were asked to take corrective actions by reevaluating their nucleic acid extraction protocols and internal quality control processes according to the laboratory guidelines (*4*,*17*) and implementing the routine use of a commercial reference material. A follow-up EQA consisting of 5 samples was conducted a month later, and clinical laboratories participated in a follow-up EQA showed all acceptable results.

For the assessment of semiquantitative data, only 0.6% outlier results were obtained by 67 laboratories using the PowerChek 2019-nCoV kit. In contrast, 4.9% outlier results were reported from 38 laboratories using the Allplex nCoV 2019-nCoV kit. For the PowerChek 2019-nCoV kit, which uses 2 PCR tubes, 2 separate tubes for both the *E* and *RdRp* genes are necessary per sample, and the volume of RNA is 5 μL per reaction tube (*4*,*17*). For the Allplex 2019-nCoV kit, the internal control is directly added into the sample, and the volume of RNA is 8 μL. The target genes are the *E*, *RdRp*, and *N* genes. The manufacturers’ reported limit of detection for the *E* gene is 28.5 copies/reaction for the PowerChek 2019-nCoV kit and 100 copies/reaction for the Allplex 2019-nCoV kit. Whether the lower limit of detection and decreased multiplexing (each gene and an internal control per tube for the PowerChek 2019-nCoV kit vs. 3 target genes and an internal control per tube for the Allplex 2019-nCoV kit) affected the performance of the 2 reagents or whether matrix effects occurred during use of the Allplex 2019-nCoV kit requires further investigation.

The first step in nucleic acid amplification tests requires extraction and purification of nucleic acids from the target organism (*18*). All 13 laboratories using the STARMag DNA/RNA extraction kit reported >1 outliers. During the preevaluation of the extraction systems and EUA rRT-PCR assays that were performed in the central laboratory before dispatch, a combination of STARMag DNA/RNA extraction and Allplex 2019-nCoV kits showed a negative result for the *E* gene in the nCoV-20–41 specimen, which had the lowest viral load. Specimen viscosity and higher rates of PCR inhibition account for sputum being the most difficult specimen type to analyze in the laboratory (*19*). Although the manufacturer of the STARMag DNA/RNA extraction kit claims that sputum and bronchoalveolar lavage are suitable types of specimens, laboratories should verify these claims and assess the performance of nucleic acid amplification tests when using this reagent for extracting RNA from lower respiratory tract samples. The laboratories given incorrect qualitative results were requested to compare their nucleic acid extraction system with the QIAamp Viral RNA Mini kit (QIAGEN, https://www.qiagen.com) (*17*).

All EQA samples were adequately homogeneous and stable on storage at −70°C. Because the complete process of this EQA, from proficiency test panel preparation, panel freezing, and logistics to completion of testing and result reporting, was finished within a week, the assessment was conducted in 7 days. The reliability of the virus panels used in the EQA was found to be stable.

Our study has some limitations. First is the small number of negative SARS-CoV-2 samples to evaluate cross-contamination, including only 2 negative samples. More negative samples placed adjacent to the highest SARS-CoV-2 sample should be included in future EQA for the evaluation of cross-contamination. Second, the potential matrix effect of the additives to the lower respiratory tract samples was not evaluated properly. Thus, for the laboratories using the Allplex 2019-nCoV kit, the performance of the reagent when using lower respiratory tract samples might not have been assessed adequately. Because variations occurred in the tested specimen types, RNA extraction platforms, PCR reagents and amplification platforms, and the amount of RNA used in the PCR reaction, many potential variables could have affected the results. Third is the small volume of EQA samples. A sufficient amount of sample for certification of multiplex technicians is recommended for certain EQA exercises; however, EQA samples were enough for »4 test runs when using a QIAamp Viral RNA Mini kit. Fourth, the EQA panel had limitations regarding test consistency evaluation; measured values varied between sample types because of a potential matrix effect, despite having the same viral loads from both upper and lower respiratory samples. An international EQA that includes replicate samples for consistency evaluation is ongoing, and the laboratories conducting SARS-CoV-2 testing are highly encouraged to participate in that program.

In conclusion, this report summarizes the nationwide EQA of SARS-CoV-2 molecular testing carried out by public and nongovernmental laboratories. The observation that 6.8% of laboratories reported false-negative results shows room for improvement. Laboratories with deficiencies were requested to take additional corrective actions and then participated in a follow-up EQA. This study indicates that EQAs should be performed for all laboratories involved in COVID-19 diagnostic testing on a regular basis for evaluation of potential weaknesses in SARS-CoV-2 molecular testing procedures. This action will help to increase the quality of results. The EQA methodology used in this study will also help other countries to evaluate their own assays for SARS-CoV-2 testing.

Appendix Additional information about nationwide external quality assessment of SARS-CoV-2 molecular testing, South Korea.
