# Supplementary material for: Nationwide External Quality Assessment of SARS-CoV-2 Molecular Testing, South Korea
Source: Emerg Infect Dis. 2020 Oct;26(10):2353–60. doi: 10.3201/eid2610.202551 (PMC7510727; doi:10.3201/eid2610.202551)
Supplement: Appendix — Additional information about nationwide external quality assessment of SARS-CoV-2 molecular testing, South Korea. [file 20-2551-Techapp-s1.pdf]

Article DOI: https://doi.org/10.3201/eid2610.202551

Nationwide External Quality Assessment of SARS-CoV-2 Molecular Testing, South Korea

Appendix

Appendix Table 1. Acceptable results for SARS-CoV-2 tests by gene-based evaluation from PowerChek 2019-nCoV kit (Kogene Biotech) and Allplex 2019-nCoV kit (Seegene), Republic of Korea, March 23-March 27, 2020*

Sample	PowerChek (n = 67), no (%)		Allplex (n = 38), no. (%)†		
no.	*E* gene	*RdRp* gene	*E* gene	*RdRp* gene	*N* gene
#41	NA	NA	NA	NA	NA
#42	67 (100)	67 (100)	36 (97.3)	37 (100)	37 (100)
#43	67 (100)	67 (100)	29 (78.4)	34 (91.9)	36 (97.3)
#44	67 (100)	67 (100)	37 (100)	37 (100)	37 (100)
#45	67 (100)	67 (100)	31 (83.8)	36 (97.3)	37 (100)
#46	67 (100)	67 (100)	38 (100)	38 (100)	38 (100)
#47	67 (100)	67 (100)	38 (100)	38 (100)	38 (100)
#48	67 (100)	67 (100)	38 (100)	38 (100)	38 (100)
#49	67 (100)	67 (100)	38 (100)	38 (100)	38 (100)
#50	67 (100)	67 (100)	38 (100)	38 (100)	38 (100)

*NA, not assessed.
†For nCoV-20–42–45, the number of laboratories was 37.

Appendix Table 2. Outliers for each gene by the double-sized Grubbs test, and negative results for positive control samples or Ct values in negative samples, Republic of Korea, March 23-March 27, 2020

Sample	PowerChek 2019-nCoV (n = 67), no (%)		Allplex 2019-nCoV (n = 38), no (%)*			Standard M nCoV Real-Time Detection (n = 6), no (%)	
no.	*E* gene	*RdRp* gene	*E* gene	*RdRp* gene	*N* gene	*E* gene	*ORF1ab*
#41	2 (3.0)†	0 (0)	20 (54.1)†	9 (24.3)†	4 (10.8)†	2 (33.3)†	2 (33.3)†
#42	0 (0)	1 (1.5)	1 (2.7)†	0 (0)	0 (0)	0 (0)	0 (0)
#43	0 (0)	0 (0)	8 (21.6)†	3 (8.1)†	1 (2.7)†	0 (0)	0 (0)
#44	1 (1.5)‡	0 (0)	0 (0)	0 (0)	0 (0)	0 (0)	0 (0)
#45	0 (0)	1 (1.5)	6 (16.2)†	1 (2.7)†	0 (0)	0 (0)	0 (0)
#46	0 (0)	0 (0)	1 (2.6)	0 (0)	0 (0)	0 (0)	0 (0)
#47	1 (1.5)‡	0 (0)	0 (0)	0 (0)	0 (0)	0 (0)	0 (0)
#48	1 (1.5)	1 (1.5)	1 (2.6)	0 (0)	0 (0)	0 (0)	0 (0)
#49	0 (0)	0 (0)	0 (0)	0 (0)	0 (0)	0 (0)	0 (0)
#50	0 (0)	0 (0)	0 (0)	0 (0)	0 (0)	0 (0)	0 (0)
Total	5 (0.7)	3 (0.4)	37 (9.9)	13 (3.5)	5 (1.3)	2 (33.3)	2 (33.3)

*For nCoV-20–42–45, the number of laboratories was 37.
†Negative results were reported for these samples.
‡These samples showed Ct values of ≥35 and were interpreted as negative according to the manufacturer’s guidance.
